# Epidemiology of Human Parvovirus 4 Infection in Sub-Saharan Africa

**DOI:** 10.3201/eid1610.101001

**Published:** 2010-10

**Authors:** Colin P. Sharp, Marion Vermeulen, Yacouba Nébié, Cyrille F. Djoko, Matthew LeBreton, Ubald Tamoufe, Anne W. Rimoin, Patrick K. Kayembe, Jean K. Carr, Annabelle Servant-Delmas, Syria Laperche, G.L. Abby Harrison, Oliver G. Pybus, Eric Delwart, Nathan D. Wolfe, Andrew Saville, Jean-Jacques Lefrère, Peter Simmonds

**Affiliations:** Author affiliations: University of Edinburgh, Edinburgh, Scotland (C.P. Sharp, P. Simmonds);; South African National Blood Service, Weltevreden Park, South Africa (M. Vermeulen, A. Saville);; Centre National de Transfusion Sanguine, Ouagadougou, Burkina Faso (Y. Nébié);; Global Viral Forecasting Initiative, Yaounde, Cameroon, and San Francisco, California, USA (C.F. Djoko, M. LeBreton, U. Tamoufe, N.D. Wolfe);; University of California School of Public Health, Los Angeles, California, USA (A.W. Rimoin);; Kinshasa School of Public Health, Kinshasa, Democratic Republic of the Congo (P.K. Kayembe);; University of Maryland School of Medicine, Baltimore, Maryland, USA (J.K. Carr);; Institut National de la Transfusion Sanguine, Paris, France (A. Servant-Delmas, S. Laperche, J.-J. Lefrère);; University of Oxford, Oxford, UK (G.L.A. Harrison, O.G. Pybus); Blood Systems Research Institute, San Francisco (E. Delwart);; Stanford University, Stanford, California, USA (N.D. Wolfe);; Centre Hospitalier Universitaire, Amiens, France (J.-J. Lefrère)

**Keywords:** Viruses, human parvovirus, PARV4, parenteral transmission, HIV/AIDS and other retroviruses, hepatitis C virus, blood donor, expedited, sub-Saharan Africa, dispatch

## Abstract

Human parvovirus 4 infections are primarily associated with parenteral exposure in western countries. By ELISA, we demonstrate frequent seropositivity for antibody to parvovirus 4 viral protein 2 among adult populations throughout sub-Saharan Africa (Burkina Faso, 37%; Cameroon, 25%; Democratic Republic of the Congo, 35%; South Africa, 20%), which implies existence of alternative transmission routes.

Human parvovirus 4 (PARV4) was originally detected in plasma from a person at risk for infection with HIV through injection drug use (*1*). Genetic characterization of the complete genome sequence of the virus showed a distant relationship to existing genera within the family *Parvoviridae*, although viruses showing 61%–63% sequence similarity to PARV4 have recently been described in pigs and cows (*2*), together likely meriting the designation of a new genus within the family. Although infections with PARV4 are not followed by long-term viremia, viral DNA sequences can likely be detected in tissues lifelong after exposure (*3**–**6*), a form of latency or persistence shared with other human parvoviruses, e.g., human parvovirus B19, and adeno-associated viruses (*6**–**8*).

PARV4 differs strikingly from other parvoviruses in its epidemiologic associations and inferred routes of transmission. Initial studies of autopsy tissue demonstrated high DNA detection frequencies among injection drug users co-infected with hepatitis C virus (HCV) in the United Kingdom, Italy, and Germany (*3**–**5**,**9*). Infection frequencies were higher in those who were HIV seropositive but almost absent in low-risk, HCV-negative and HIV-negative control populations. Despite these new insights, studies based on autopsy or biopsy tissues are cumbersome and necessarily limited by sample availability and technical complexity.

## The Study

To address gaps in knowledge about PARV4, we have recently developed an ELISA for antibodies to the viral protein 2 (VP2) of PARV4 genotype 1 to expand investigations of epidemiology and transmission of the virus (*10*). Larger scale screening confirmed the previously noted association between PARV4 infection and parenteral routes of exposure (injection drug use) in the United Kingdom and United States, much lower infection frequencies in HIV-infected gay men, and zero seropositivity in low-risk controls. We additionally found serologic evidence for high rates of PARV4 exposure among persons with hemophilia exposed to nonvirally inactivated factor VIII/IX concentrates but a virtual absence of infection in sibling controls occupying the same households.

To investigate further the epidemiology of PARV4 in sub-Saharan Africa, we assembled large sets of serum or plasma samples collected from a range of adult populations in several countries in Africa (Table). Samples were screened in duplicate by our previously described ELISA (*10*) by using protein purified in parallel from empty baculovirus constructs as control antigen to minimize assay nonspecificity. Low-risk orthopedic outpatient attendees (United Kingdom) and HIV-negative and HCV-negative nonremunerated blood donors (France) were used as negative control populations.

**Table:** Seroprevalence of human parvovirus 4 antibodies in sub-Saharan African and control populations*

Country	Category	No.	Co-infection		Mean year of birth (range)	Collection year	PARV4 positive, no. (%)
HIV†	HCV‡
Burkina Faso	Blood donors	167	0	0	1982 (1951–1999)	2007	62 (37.1)
Cameroon	General population	238	0	0	1968 (1962–1972)	2007	59 (24.8)
Democratic Republic of the Congo	Military population	221	2§	0	1968 (1936–1986)	2007	78 (35.3)
South Africa	Blood donors (HIV-positive)	170	170	0	1976 (1945–1990)	2007	62 (36.4)
South Africa	Blood donors (HIV-negative)	180	0	0	NA	2009	8 (4.4)
United Kingdom	General population	161	ND	ND	1950 (1937–1977)	2005	0
France	Blood donors	199	0	0	1965 (1943–1989)	2008	0

*HCV, hepatitis C virus; PARV4, human parvovirus 4; NA, not available; ND, screening not done. †HIV-1 screening methods: South Africa, France: fourth-generation ELISA. ‡HCV screening methods: Burkina-Faso, South Africa, France: third-generation ELISA, recombinant immunoblot assay confirmation of positive results; Democratic Republic of the Congo: third-generation ELISA, exclusion of reactive samples; Cameroon: PCR-based screening, exclusion of PCR-positive samples. United Kingdom: not screened (ND), low risk background and absence of parenteral or HIV risk factors. §1 of 2 HIV-positive samples was seropositive for PARV4.

Serologic screening for PARV4 antibodies showed that the combined set of 360 blood donor and control samples from the United Kingdom and France were nonreactive by ELISA (Table). In marked contrast, high rates of anti-PARV4 reactivity were detected in populations from sub-Saharan Africa. The highest rates were observed in Burkina Faso, where a frequency of 37% was recorded among a screened HIV-negative and HCV-negative blood donor population. Frequencies of seropositivity were 25% and 35% in Cameroon and Democratic Republic of the Congo, respectively, and lowest in South Africa (4% in HIV-negative persons). However, within the latter group, HIV-1–infected donors were significantly more frequently seropositive for PARV4 than those who were not infected with HIV (36%; p<0.0001 by Fisher exact test). However, even with this risk factor, the overall prevalence was not as high as observed in the HIV-negative blood donors in Burkina Faso.

## Conclusions

These findings provide new and unexpected information on the epidemiology and transmission of PARV4. First, although there is no evidence of PARV4 infection in nonpotentially exposed persons in Western countries (from the limited number currently surveyed), populations in sub-Saharan Africa, particularly in Central Africa, show high rates of exposure that cannot plausibly be accounted for by parenteral exposure. For example, the highest rate of seropositivity was observed among blood donors in Burkina Faso and Democratic Republic of the Congo who were uniformly negative for HCV antibodies, as well as for HIV-1 antibodies, by third-generation screening. In this setting, HCV infections are a frequent correlate of multiple blood transfusions and use of unsterilized needles in medical treatment or vaccination, as well as injection drug use. The high rate of seropositivity among HCV screen-negative samples from all 4 countries in Africa provides strong evidence for an alternative route of PARV4 transmission that is largely or entirely absent in Western countries.

These findings are consistent with PCR-based evidence for PARV4 viremia, presumably associated with acute infection, among young children in rural Ghana (*11*), a country adjacent to Burkina Faso where similar conditions for virus transmission may exist. In this study group, parenteral exposure was not identified, although infections were more frequent in families in low socioeconomic groups and those living near rivers and without a domestic water supply. In a separate study, autopsy samples from 2 HIV-infected African men (from Nigeria and Democratic Republic of the Congo) were PARV4 positive, despite not being infected with HCV and without a history of parenteral exposure (*12*). Neither study identified the specific risk factors and PARV4 infection sources.

It clearly is a challenge to conceive transmission routes for a virus that will not be directly transmitted among members of the same household, as demonstrated by the hemophiliac sibling data for the United States (*10*). Hypotheses such as possible arthropod-borne or parasite-associated transmission require careful evaluation, bearing in mind that parvoviruses, in common with other DNA viruses, are highly host species specific, and no other instances of vector-borne transmission in this virus family have been recorded.

The second major observation was a substantial difference in the rate of PARV4 seropositivity between those who were HIV infected and those uninfected (in South Africa, 36% and 4%, respectively). This association with HIV appears initially consistent with higher frequencies of PARV4 seropositivity reported among HIV (and HCV) co-infected injection drug users in Western countries. However, in the latter group, the association with HIV-1 was thought to reflect a greater frequency of illicit injection with shared needles (*4**,**9*). Furthermore, low frequencies or absence of PARV4 infections were observed in HCV-uninfected persons acquiring HIV-1 infection by sexual contact. How HIV-1 facilitates or becomes epidemiologically associated with PARV4 infection in HCV-negative South African blood donors thus remains unexplained in a population where HIV-1 infections are primarily acquired through sexual contact.

Although this study leaves many questions on the transmission of PARV4 unanswered, the striking differences in seroprevalence and risk-group associations between sub-Saharan Africa and Western countries provides the basis for future more detailed investigations of its transmission routes, epidemiology, and potential clinical outcomes of infections. The previously noted sequence homogeneity of PARV4 nucleotide sequences between variants detected in Western countries (*4**,**13*) is consistent with its recent global spread. A possible source in sub-Saharan Africa for PARV4 would contain many potential parallels with the emergence and global spread of HIV-1 and HCV in the 20th century.
